# Exploring Food Insecurity and Perceived Stress on Daytime Sleepiness among Older Adults in New York City

**DOI:** 10.3390/foods13172831

**Published:** 2024-09-06

**Authors:** Collette Brown, John Orazem, Elgloria Harrison

**Affiliations:** School of Health Sciences, Human Services, and Nursing, Lehman College, City University of New York, Bronx, NY 10468, USA; john.orazem@lehman.cuny.edu (J.O.); elgloria.harrison@lehman.cuny.edu (E.H.)

**Keywords:** food insecurity, food security, perceived stress, sleepiness, sleep quality

## Abstract

The growing population of older adults in the U.S. is experiencing increased food insecurity and stress, which are associated with nocturnal sleep quality and consequently excessive daytime sleepiness. This study aimed to investigate the relationship between food insecurity and perceived stress on daytime sleepiness in older adults aged 60 and older living in New York City. This cross-sectional, quantitative study utilized the US Household Food Security Survey Module six-item questionnaire, the Perceived Stress Scale (PSS), and Sleepiness Total to collect data. Participants completed an online survey via Qualtrics. Linear and logistic regression models were used to assess the association between demographic variables and food insecurity, perceived stress, and daytime sleepiness outcomes. Three hundred seventy-eight (378) older adults participated in this study. Food insecurity was associated with age (*p* = 0.045), education (*p* = 0.022), and daytime sleepiness (*p* < 0.001). On average, participants with a BMI of over 30 had an increased daytime sleepiness total relative to a BMI < 25 (*p* = 0.029), and those with two to three health conditions and those with more than four health conditions had higher daytime sleepiness totals relative to those with zero to one condition (*p* = 0.007 and 0.007, respectively). Participants who had moderate and high stress, regardless of food security status, had higher daytime sleepiness totals than those with low stress (food secure; *p* = 0.002; food insecure; *p* < 0.001). Multifaceted interventions are needed to alleviate food insecurity, manage stress, and reduce excessive daytime sleepiness among older adults.

## 1. Introduction

The population of older adults over 60 years was 78 million in 2021 [1]. As this population ages, there is an increased vulnerability to developing chronic diseases, many of which are linked to poor nutrition. Factors such as poor nutritional intake, lack of physical activities, social isolation, and fear of the loss of autonomy further complicate the aging process of older adults [2]. Compounding these issues is the increased prevalence of food insecurity, which poses a particularly severe challenge among older adults.

FI is defined as inconsistent access to sufficient, safe, and nutritious food, or the limited ability to obtain food in socially acceptable ways for an active, healthy life among all members of a household [3,4]. The prevalence of FI among adults aged 60 years and older was 7.1% in 2021 [1]. FI among older adults has steadily increased since 2020. Between 2020 and 2022, the prevalence of food insecurity in U.S. households with at least one older adult aged 65 or older was reported to be of 6.9%. 7.1%, and 9.1%, respectively [3]. FI is more prevalent among older adults who are Black or Hispanic, those aged 60–69 years, individuals with low incomes [1,5], those who have a high school education or less [5], and those who live in rented homes [1]. Given this information, addressing food insecurity among older adults is crucial to ensuring long-term health and quality of life.

FI is a public health concern among older adults in the United States, posing significant challenges to their health and well-being. It has been linked to worse health outcomes and poor sleep quality [6,7]. Research suggests that many older adults face challenges in securing adequate and nutritious foods [7,8,9,10], which may increase negative health outcomes [7,11,12,13,14,15,16,17,18,19,20] and increase health services utilization [9]. Some of these challenges include acute or chronic illnesses, health complications, distance from food sources, inadequate transportation, affordability of foods, low income, separation or divorce, unemployment, and living alone [8]. These challenges are associated with food insecurity, causing individuals to have imbalanced meals, skip meals, feel hungry without the finances to purchase food, or eat less food overall [9]. Moreover, older adults generally have restricted calorie intake due to changes in their metabolism, age-related bone and muscle loss, and an increased sedentary lifestyle [10]. Food insecurity may be associated with worsening psychological health consequences, such as cognitive decline [7,11,12], anxiety, depression, perceived stress [13,14,15,16,17,18,19,20], and sleep problems [6,21,22,23,24]. For example, when individuals are food insecure, they tend to worry about their next meal, they may experience reduced food intake, or possibly skip meals. Subsequently, these factors may lead to stress and lack of nighttime sleep, which may prompt daytime sleepiness. FI is associated with greater odds of having chronic conditions such as asthma and diabetes [14] as well as obesity, heart disease, and pulmonary diseases [25]. Individuals who are food insecure may also have unhealthy dietary patterns and limited fruit and vegetable intake [26]. These cumulative effects may also lead to the increased utilization of health services for adverse health conditions [9] and lower health-related quality of life [16,25]. Therefore, access to adequate and nutritious foods is necessary for the optimal health of older adults.

While FI is a big concern for older adults, so is the notion of perceived stress. Perceived stress refers to the extent to which a person perceives life’s events as stressful [27]. Previous studies have reported that perceived stress is associated with excessive daytime sleepiness [28], which is defined as difficulty staying awake or alert, or an increased desire to sleep during the day in situations wherein wakefulness is expected [29]. In older adults, common sleep disturbances may be associated with excessive daytime sleepiness [30]. Insufficient nighttime sleep is associated with excessive daytime sleepiness [31], inadequate daytime alertness, disruption of daily activities, diminished physical activities, increased risks of accidents, social incompatibility, poor health outcomes, and poor quality of life in older adults [30]. Daytime sleepiness may result in motor vehicle and industrial accidents, decreased productivity, interpersonal problems [32], and falls [33]. Food insecurity, stress, and excessive daytime sleepiness are modifiable risk factors for many chronic diseases in older adults.

Despite the importance of food insecurity and daytime sleepiness among older adults, few studies have addressed their associations among this population. For example, some of the studies either focus on nighttime sleep [18] or a combination of nighttime sleep and naps during the day to determine the total number of hours a person sleeps in a day [6,17]. In addition, Ref. [19] conducted a study on food insecurity which took into account the duration of sleep and sleep disorders using a working-age adult sample (22–60 years). To the best of our knowledge, no research has been conducted to investigate the impacts of food insecurity and daytime sleepiness among older adults (65 years and older in the U.S.). Therefore, the purpose of this study was to assess the associations between food insecurity and perceived stress on daytime sleepiness, as well as the relationship between demographics and daytime sleepiness, food security, and perceived stress, among older adults in New York City.

## 2. Materials and Methods

### 2.1. Sample and Study Design

This study utilized a cross-sectional, quantitative study design to collect data from 378 participants, who were 60 years and older and lived within the five boroughs of New York City (NYC). Data were collected between August 2023 and January 2024, using an online survey that was distributed through Qualtrics (Provo, UT, USA). Qualtrics is an online marketing research organization that managed the recruitment process using specifications provided by the authors. Qualtrics sought to obtain a sample that was balanced by sex (male, female) and a combination of race (White, Asian, Black) and ethnicity (Hispanic, Non-Hispanic). Participants read the informed consent and answered the question “Do you want to participate in this survey?” If they answered “yes”, they were allowed to proceed to answer the survey.

### 2.2. Measures

Demographics: Demographic, socioeconomic, and health data were collected from the participants, including age, race/ethnicity, gender, household income, education, BMI, and number of health conditions. Body Mass Index (BMI): Participants were asked to self-report and record their weight and height to calculate their BMI. BMI was calculated as (pounds/inches^2^) × 703, where individuals who had BMIs < 18.5 were considered underweight, 18.5–24.9 were considered to be normal BMIs, 25–29.5 were considered overweight, and 30 and above were considered obese [34]. Chronic Conditions: Participants were asked to self-identify individual health conditions (e.g., diabetes). For this study, the number of reported health conditions was grouped as 0–1, 2–3, and 4 or more health conditions.

Daytime Sleepiness Total: To measure daytime sleepiness, we used seven questions from the Epworth Sleepiness Scale (ESS), which was developed by [35] and is a validated instrument that was used to assess the participants’ subjective, self-reported daytime sleepiness. The ESS consists of eight questions that ask respondents to rate their chances of dozing off or falling asleep in certain situations. Some situations include watching TV, sitting and reading, and sitting and talking to someone. Responses were measured on a four-point scale ranging from 0 to 3, where 0 = would never doze, 1 = slight probability of dozing, 2 = moderate chance of dozing, and 3 = high likelihood of dozing. Higher daytime sleepiness scores indicate higher average sleep propensity (ASP) in daily life, or their “daytime sleepiness”. A total based on 7 of the 8 items was used for this study (the item “sitting quietly after a lunch without alcohol” was inadvertently omitted from the survey); however, the 7-item total retained good psychometric internal consistency: Cronbach’s alpha = 0.76 [36].

Food Security Scale: Food insecurity (FI) was assessed using the US Household Food Security Survey Module six-item questionnaire developed by the US Department of Agriculture, Economic Research Service [37]. This short-form six-item scale is a subset of the standard 18-item questionnaire and is a reasonably reliable substitute [3]. It has high specificity and sensitivity in measuring food-insecure and very food-insecure households [37]. The survey contained questions about the availability of food eaten in participants’ households in the last 12 months, and whether they were able to afford the food that they needed. Participants responded to statements such as “In the last 12 months, did you ever eat less than you felt you should because there wasn’t enough money for food?” and “In the last 12 months, were you ever hungry but didn’t eat because there wasn’t enough money for food?” Responses to these questions were yes, no, and don’t know. Other questions included “The food that (I/we) bought just didn’t last, and (I/we) didn’t have money to get more.” Responses were often, sometimes, or never true for (you/your household) in the last 12 months. Scores relating to each question on food security status were summed and calculated on a scale of 0 to 6. Scores between 0 and 1 indicated high or marginal food security; scores between 2 and 4 indicated low food security, and scores between 5 and 6 indicated very low food security (VLFI). The responses were dichotomized into food security (high or marginal food security) and food insecurity (low or very low food insecurity).

Perceived Stress Scale: Perceived stress was measured using the 10-item Perceived Stress Scale (PSS), which is a reliable and commonly used instrument to assess the frequency of a person’s stress perception in the last month [38]. Participants scored their emotional and cognitive responses to specific situations in their daily lives. The questions are scored on a 5-point Likert scale ranging from 0 to 4, where 0 = never, 1 = almost never, 2 = sometimes, 3 = often, and 4 = very often. The scores on the instrument range from 0 to 40. Scores ranging from 0 to 13 were considered low stress, scores from 14 to 26 were considered moderate stress, and scores from 27 to 40 were considered high stress [38].

### 2.3. Data Analysis

Participant characteristics were summarized using cell counts and percentages; these characteristics included demographics (including BMI categories and number of health conditions), Food Security Status, and categorized versions of Sleepiness Total and Perceived Stress Total. The categorization of Sleepiness Total was based on quintiles, and the categories for Perceived Stress Total were taken from guidance from the New Hampshire Employee Assistance Program [38]. The Sleepiness Total was analyzed using a linear regression model that included all demographic variables as covariates; the statistical tests (overall F test and pairwise comparisons to a reference category) of each demographic variable were adjusted for the other demographics; the Perceived Stress Total was analyzed similarly. Food Security Status was analyzed similarly, except a logistic regression model was used and only the overall chi-squared test was calculated. The Sleepiness Total was further analyzed using a 2-way factorial ANCOVA interaction model, wherein the Food Security Status and Perceived Stress categories were treated as factors, and the demographic variables were included as covariates. The bivariate relationship between Sleepiness Total and Perceived Stress Total was explored using Pearson correlation and a linear regression model predicting Sleepiness Total as a function of Perceived Stress Total. As this study was exploratory, no adjustment for multiple comparisons was planned or implemented. No imputations of missing data were used; only observed data were analyzed. *p*-values < 0.05 were considered statistically significant. All analyses were conducted using R version 4.3.1 [39]. The regression models underlying summaries in tables and figures are detailed in the Supplemental Tables.

## 3. Results

### 3.1. Sample Characteristics

Three hundred and seventy-eight (378) individuals aged 60 years and older participated in this study. Table 1 shows that 47.5% were between the ages of 60 and 65 years and most of the participants were non-white (51.5%), earned less than USD 75,000 (70.7%), had less than a bachelor’s degree (53.7%), were overweight or obese (64.1%), and had two or more health conditions (66.2%). Among the older adults participating in this study, 45.7% experienced low levels of daytime sleepiness (total score 0–4) and 33.8% experienced excessive daytime sleepiness (total score of 7 or greater). Food insecurity was classified as having low or very low food security; the prevalence of food insecurity among older adults was approximately 19.1%. Of the 378 participants, the prevalence of perceived stress was 49.2%, 45.8%, and 5.0% for low, moderate, and high stress, respectively.

### 3.2. Association between Daytime Sleepiness and Demographic Variables

Table 2 presents pairwise comparisons to the reference category (first category) for each variable, as well as the overall *p*-value. There was a statistically significant difference between obese participants versus normal/underweight participants for the tendency to become sleepy (*p* = 0.029), wherein obese participants were more likely to experience daytime sleepiness (mean = 6.22 for obese versus mean = 4.63 for normal/underweight). In addition, concerning daytime sleepiness total, there was a statistically significant difference between participants reporting two to three health conditions versus those reporting zero to one condition (*p* = 0.007), and similarly for those reporting four or more health conditions (*p* = 0.013). In each comparison to those with the least number of health conditions, the participants with more health conditions were more likely, on average, to experience daytime sleepiness. There were no statistically significant differences in age, gender, race/ethnicity, income, or education regarding daytime sleepiness (Table 2).

### 3.3. Analysis of Food Insecurity (FI) Categories by Demographics

Analyses of the association between FI categories and demographic variables are shown in Table 3. There was a tendency for food security to increase with age (*p* = 0.045). Older participants (older than 75 years) tended to be more food secure (94.1%) compared to participants between 60 and 65 years old (73%). The association with education was also statistically significant (*p* = 0.022). Participants with a high school education or less were more food secure (84.4%) than any other educational group.

### 3.4. Analysis of Perceived Stress Total by Demographics

Table 4 presents pairwise comparisons to the reference category (first category) for each variable, as well as the overall *p*-value. There were no statistically significant associations between demographic variables and the perceived stress total.

### 3.5. Factorial Analysis of Sleepiness Total by Food Security Status and Perceived Stress Category

Table 5 summarizes a two-way factorial analysis of sleepiness total by the food security and perceived stress factors, adjusted for the demographic variables. As this table shows, there was a tendency, evident within each food security category, for daytime sleepiness to increase as perceived stress increased (overall *p* < 0.001). Also, within each of the moderate stress and high-stress categories, the very low/low food security category scored higher on the sleepiness total, on average, relative to the high/marginal food security category (*p* < 0.001).

### 3.6. Correlation between Perceived Stress and Daytime Sleepiness

**Figure 1:**
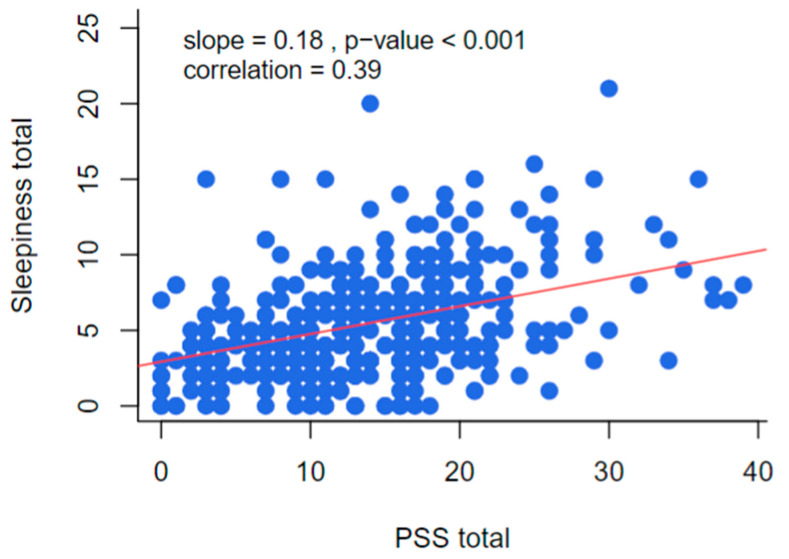
Correlation between perceived stress and daytime sleepiness.

The correlation between perceived stress total and daytime sleepiness total is presented in Figure 1. This correlation was moderate in magnitude and positive in direction, meaning that, as perceived stress levels increase, the sleepiness scores tend to increase (correlation coefficient = 0.39; *p* <0.001).

## 4. Discussion

We were particularly interested in food security, perceived stress, and their associations with daytime sleepiness among older adults in New York City. Our study revealed that older adults who were food insecure tended to report greater daytime sleepiness compared to those who were food secure (*p* = 0.043 for food security from factorial analysis). Being food insecure may lead to restricted caloric intake, where hunger may contribute to poor sleep quality at night [20]. Consequently, individuals may experience more daytime sleepiness and take more naps during the day. The results of this study are like those of the previous study [19], wherein food-insecure participants reported more sleepiness and perceived less control over their sleep. These findings may help to ensure that older adults are informed about food assistance programs in their communities.

Food insecurity has become a public health concern among older Americans and, as this research suggests, the prevalence of food insecurity among older adults participating in our study was approximately 19%. Although not presented in the tables, about 12% of participants in our study had very low food insecurity, compared to the national average of 9.1% in 2022 [3]. We found a statistically significant inverse relationship between FI and age (*p* = 0.045), wherein older participants (75 years and older) were more likely to be food secure compared to participants who were 60–65 years old. Our study aligns with the results from a previous study conducted in the U.S. [16]. The younger participants may have more financial responsibilities (mortgage payments, children in college, etc.) compared to older participants over 75 years.

Additionally, African Americans and Hispanics were two times more likely to experience FI compared to Whites, but this tendency was not statistically significant. Regardless of age, numerous studies have indicated that food insecurity is higher in Black and Hispanic adults compared to White adults [5,16,17]. For example, Ref. [5] indicated that the prevalence of food insecurity among non-Hispanic Black and Hispanic adults was almost two times higher than among Whites. Furthermore, our research revealed that individuals with a high school education or less were more likely to experience food security, whereas those with some college education but without a bachelor’s degree were less likely to be food secure. Our results differ from previous research [16,17,21]. For example, Ref. [21] mentioned that the odds of individuals living in food-insecure households were higher for individuals with lower education. A possible explanation for our results could be that the individuals with high school or less education may be participating in various assistance programs such as the Supplemental Nutrition Assistance Program (SNAP) and food pantries to supplement their food intake.

A growing body of literature indicates that food insecurity is associated with chronic health conditions [14,21]; therefore, increasing health service utilization [9]. However, contrary to these studies, our study did not indicate an association between food insecurity and the number of health conditions among older adults. A possible explanation for the difference in results could be that previous studies may have separately analyzed food insecurity with one outcome variable at a time, for example, diabetes or hypertension, while this current study analyzed food insecurity based on having one or more chronic conditions together.

Our study results indicate that, regardless of food security status, older adults who had moderate and high stress were more likely to have higher daytime sleepiness totals compared to those who had low perceived stress levels. In a study conducted by [40], they found that older adults experiencing frequent stress co-occurrence had difficulty initiating sleep, and were more likely to experience frequent nocturnal awakening and nonrestorative sleep. These stressors may include securing nutritious foods, managing health conditions, and dealing with socioeconomic and political conditions. Worrying about how to access food, especially for someone with multiple chronic conditions, or having to prioritize paying rent over purchasing food due to low finances, may contribute to inadequate sleep at night [9]. When individuals do not sleep well or have an inadequate amount of nighttime sleep, they tend to be sleepier during the day [41,42]. Therefore, recognizing and reducing sources of stressors is important because increased stress can affect both sleep quality and duration of sleep among older adults.

In this current study, excessive daytime sleepiness was associated with a BMI greater than 30. A previous study indicates that participants who were obese (BMI > 30) had higher sleepiness scores compared to those who had normal BMIs [43]. In their study, the researchers used the Epworth Sleepiness Scale to measure daytime sleepiness. These studies indicate that obese individuals may sleep during the day even if they slept well during the night. In addition, our study revealed that daytime sleepiness was related to an increased number of health conditions. A review article, Ref. [44], reported that several neurological, psychological, and cardiopulmonary conditions may contribute to excessive daytime sleepiness. Therefore, clinicians must diagnose individuals with excessive daytime sleepiness and address its underlying risk factors.

There were a couple of limitations to this research. Firstly, the fact that it was only available online may have introduced a technology bias, as not everyone has access to computers. To mitigate this, the survey was designed to be completed on smartphones. Secondly, there may have been recall bias among participants, especially among older adults, as they may have had difficulty accurately recalling past events. This could have affected the accuracy of their responses. The third limitation was the self-reporting of participants’ heights and weights, which could introduce bias in the study. The fourth limitation is the study’s cross-sectional design, where data were collected at a single point in time. The exposure and outcome variables were analyzed simultaneously; therefore, a cause-and-effect relationship could not be established. Finally, our population included older adults from New York City. Therefore, the results cannot be generalized beyond the study population.

## 5. Recommendations and Conclusions

Policy, Systems, and Environmental (PSE) changes are approaches that can be implemented to address the effects of food insecurity, perceived stress, and sleepiness among older adults. These approaches are separate but can be complementary in providing multifaceted interventions among communities in which older adults reside. According to [45], older adults are an engaged section of the population, particularly those associated with the baby boomer age group (individuals who were born between 1946 and 1964). This age group tends to be healthier, more educated, politically active, and avid consumers of healthcare services [45]. Policies that increase the likelihood that older adults are more food secure will be ideal for local, state, and national governments. Providing health services to this segment of the population may decrease the cost of institutionalized care, keep older adults in their homes, and support families in caring for older adults in their communities.

Based on the results of our study, the researchers suggest that the effects of food insecurity may be reduced by using several food assistance programs that provide low-income individuals with food. The studies [4,8] recommended SNAP, Temporary Emergency Food Assistance Program (TEFAP), Commodity Supplemental Food Program (CSFP), Child and Adult Care Food Program (CACFP), Senior Farmers’ Market Nutrition Program (SFMNP), and Home-Delivered Meals Congregate Meal Program for seniors. The authors of [46] conducted a study that showed that the expansion of SNAP eligibility to individuals who receive Social Security income (SSI) was associated with improvement in food security and general health. Since we did not verify whether our participants received food assistance in our research, we recommend that future research include questions on supplemental nutrition programs. While the provision of food assistance programs may not solve the problem of food insecurity, we believe that there is an opportunity at the local, state, and national levels to improve food security in older adults.

Recognizing the relationships between food insecurity and perceived stress on daytime sleepiness, we recommend advocating for policies that will broaden insurance coverage to include comprehensive sleep evaluations, treatment for sleep disorders, and improved stress management services. At the community level, local governments should implement community-based stress reduction programs aimed at promoting mindfulness, relaxation techniques, and exercise programs to benefit older adults. In addition, we recommend that more efforts should be made to encourage older adults to join urban farms in their communities. Engaging in physical activities such as community gardening can serve as a therapeutic outlet, offering physical activities and opportunities for social interactions with fellow community members [47]. It could also decrease daytime sleepiness and improve longer sleep duration at night.

In conclusion, there is a growing body of research on conditions that are associated with healthy aging, and food insecurity has been rising as a public health concern among older adults. Our research investigated how food insecurity and stress have an outside influence on daytime sleepiness. We observed in our results that older adults who are food insecure and whose stress levels are amplified have higher daytime sleepiness scores. If older adults are worried about not having enough food to eat or having to skip meals, they create a vicious cycle of worry, and poor sleep quality at night, which may be associated with excessive daytime sleepiness. When older adults enter this vicious cycle, the ability to manage and make decisions about their health may be altered, leading to the potential for poor health outcomes and poor quality of life. This specific group of older adults stands to gain significantly from additional support to ensure food security, reduced stress levels, improved sleep quality, and an overall enhanced quality of life.

## Figures and Tables

**Table 1:** Summary of participants: demographics, sleepiness, food security, and perceived stress (N = 378).

Variable	Category	n (%)
Age, years (N = 358)	60–65	170 (47.5)
	66–75	148 (41.3)
	>75	40 (11.2)
Gender (N = 378)	Female	219 (57.9)
	Male	159 (42.1)
Race/Ethnicity (N = 377)	Black or African American	59 (15.6)
	Hispanic	104 (27.6)
	White	183 (48.5)
	Other	31 (8.2)
Household Income (N = 358)	<USD 15,000	45 (12.6)
	USD 15,000–USD 34,999	85 (23.7)
	USD 35,000–USD 74,999	123 (34.4)
	≥USD 75,000	105 (29.3)
Highest Degree Earned (N = 378)	High school or less	80 (21.2)
	Some college/2-year or associate’s degree	123 (32.5)
	4-year or bachelor’s degree	100 (26.5)
	Graduate degree	75 (19.8)
Body Mass Index (BMI) (N = 378)	Underweight or normal weight (<25)	136 (36.0)
	Overweight (25–29.9)	125 (33.1)
	Obese (30+)	117 (31.0)
Number of Health Conditions (N = 378)	0–1	128 (33.9)
	2–3	145 (38.4)
	4+	105 (27.8)
Daytime Sleepiness (N = 378)	0–2	75 (19.8)
	3–4	98 (25.9)
	5–6	77 (20.4)
	7–8	58 (15.3)
	9+	70 (18.5)
Food Security Status (N = 356)	High or marginal food security	288 (80.9)
	Very low or low food security	68 (19.1)
Perceived Stress Scale (N = 378)	Low stress	186 (49.2)
	Moderate stress	173 (45.8)
	High stress	19 (5.0)

**Table 2:** Analysis of sleepiness total by demographics.

Variable	Value	n	Mean	SD	*p*-Value *
Age, years (overall *p*-value = 0.577)	60–65	164	5.52	3.58	
	66–75	137	5.57	3.48	0.645
	>75	37	4.89	3.65	0.471
Gender (overall *p*-value = 0.098)	Female	187	5.22	3.49	
	Male	151	5.78	3.60	0.098
Race (overall *p*-value = 0.321)	Black or African American	58	5.62	3.72	
	Hispanic	100	6.00	3.77	0.781
	White	152	5.05	3.39	0.270
	Other	28	5.57	3.05	0.784
Household Income (overall *p*-value = 0.510)	<USD 15,000	42	5.90	3.94	
	USD 15,000–USD 34,999	80	5.36	3.96	0.559
	USD 35,000–USD 74,999	113	5.19	3.36	0.699
	≥USD 75,000	103	5.69	3.24	0.579
Highest Level of Education (overall *p*-value = 0.526)	High school or less	68	5.78	3.73	
	Some college/2 year or assoc. degree	114	5.64	3.38	0.536
	4-year or bachelor’s degree	89	5.07	3.59	0.147
	Graduate degree	67	5.40	3.60	0.513
BMI (overall *p*-value = 0.082)	Underweight or normal weight (<25)	120	4.63	3.26	
	Overweight (25–29.9)	113	5.66	3.47	0.132
	Obese (30+)	105	6.22	3.77	0.029
Number of Health Conditions (overall *p*-value = 0.011)	0–1	117	4.50	3.28	
	2–3	131	5.98	3.41	0.007
	4+	90	5.99	3.85	0.013

* *p*-value for pairwise comparison to the first category, adjusted for the other demographic variables in the table using an ANCOVA regression model that included all demographics above. Overall *p*-value is that from the overall F test of any effect of the demographic.

**Table 3:** Analysis of food insecurity categories by demographics.

Variables	Value	High or Marginal Food Security n (%)	Low or Very Low Food Security n (%)	*p*-Value *
Age, years	60–65	111 (73.0)	41 (27.0)	0.045
	66–75	110 (83.3)	22 (16.7)	
	>75	32 (94.1)	2 (5.9)	
Gender	Female	145 (80.6)	35 (19.4)	0.194
	Male	108 (78.3)	30 (21.7)	
Race	Black or African American	42 (76.4)	13 (23.6)	0.404
	Hispanic	68 (73.9)	24 (26.1)	
	White	125 (86.2)	20 (13.8)	
	Other	18 (69.2)	8 (30.8)	
Household Income	<USD 15,000	25 (69.4)	11 (30.6)	0.324
	USD 15,000–USD 34,999	57 (74.0)	20 (26.0)	
	USD 35,000–USD 74,999	88 (80.0)	22 (20.0)	
	≥USD 75,000	83 (87.4)	12 (12.6)	
Highest Level of Education	High school or less	54 (84.4)	10 (15.6)	0.022
	Some college/2 year or assoc. degree	74 (69.2)	33 (30.8)	
	4-year or bachelor’s degree	73 (86.9)	11 (13.1)	
	Graduate degree	52 (82.5)	11 (17.5)	
BMI	Underweight or normal weight (<25)	91 (79.8)	23 (20.2)	0.606
	Overweight (25–29.9)	86 (79.6)	22 (20.4)	
	Obese (30+)	76 (79.2)	20 (20.8)	
Number of Health Conditions	0–1	90 (79.6)	23 (20.4)	0.079
	2–3	100 (84.7)	18 (15.3)	
	4+	63 (72.4)	24 (27.6)	

* *p*-value from chi-squared test of association, adjusted for the other demographic variables in the table using a logistic regression model that included all demographics above. Table entries are cell counts and row percentages; the row percentages sum to 100%.

**Table 4:** Analysis of perceived stress total by demographics.

Variable	Value	n	Mean	SD	*p*-Value *
Age, years (overall *p*-value = 0.112)	60–65	164	14.21	7.49	
	66–75	137	14.55	8.26	0.784
	>75	37	11.84	6.39	0.065
Gender (overall *p*-value = 0.417)	Female	187	14.44	7.96	
	Male	151	13.65	7.43	0.417
Race (overall *p*-value = 0.334)	Black or African American	58	12.97	6.72	
	Hispanic	100	14.40	7.92	0.153
	White	152	14.31	8.08	0.069
	Other	28	14.11	7.19	0.371
Household Income (overall *p*-value = 0.742)	<USD 15,000	42	15.50	6.39	
	USD 15,000–USD 34,999	80	14.11	7.86	0.381
	USD 35,000–USD 74,999	113	14.06	7.27	0.416
	≥USD 75,000	103	13.52	8.59	0.276
Highest Level of Education (overall *p*-value = 0.458)	High school or less	68	14.22	7.22	
	Some college/2 year or assoc. degree	114	14.60	7.53	0.811
	4-year or bachelor’s degree	89	12.97	7.40	0.289
	Graduate degree	67	14.58	8.90	0.939
BMI (overall *p*-value = 0.737)	Underweight or normal weight (<25)	120	13.35	8.53	
	Overweight (25–29.9)	113	14.17	7.96	0.498
	Obese (30+)	105	14.85	6.39	0.498
Number of Health Conditions (overall *p*-value = 0.422)	0–1	117	13.50	8.91	
	2–3	131	13.96	6.87	0.801
	4+	90	15.04	7.24	0.216

* *p*-value for pairwise comparison to the first category, adjusted for the other demographic variables in the table using an ANCOVA regression model that included all demographics above. The overall *p*-value is that from the overall F test of any effect of the demographic.

**Table 5:** Factorial analysis of sleepiness total by food security status and perceived stress.

Food Security Status	Perceived Stress	n	Mean	SD	*p*-Value *
High or marginal food security	Low stress	154	4.29	2.89	
	Moderate stress	90	5.77	3.40	0.002
	High stress	9	7.22	3.42	0.002
Very low or low food security	Low stress	11	3.36	2.77	
	Moderate stress	47	7.45	3.60	<0.001
	High stress	7	8.57	3.10	<0.001

* *p*-value for pairwise comparison to the first category, using a 2-way factorial ANCOVA model with food security status and perceived stress as factors, and adjusted for the demographic variables in Table 1. *p*-value for main effect of perceived stress < 0.001, *p*-value for main effect of food security status = 0.043. *p*-value for test of interaction = 0.076.

## Data Availability

The original contributions presented in the study are included in the article/Supplementary Material, further inquiries can be directed to the corresponding author.

## Supplementary Materials

The following supporting information can be downloaded at: https://www.mdpi.com/article/10.3390/foods13172831/s1, Table S1: Analysis of sleepiness total by demographics. Table S2: Logistic Regression Analysis of Food Security categories by demographics. Table S3: Linear Regression Analysis of Perceived Stress Total by demographics. Table S4: 2-Way Factorial Food Security Status x Perceived Stress Category Analysis of Sleepiness Total, adjusting for demographic variables and Food Security Status. Table S5: Linear regression model for Sleepiness Total as a function of Perceived Stress Total.
