# Supplementary material for: Exploring Food Insecurity and Perceived Stress on Daytime Sleepiness among Older Adults in New York City
Source: Foods. 2024 Sep 6;13(17):2831. doi: 10.3390/foods13172831 (PMC11394888; doi:10.3390/foods13172831)
Supplement: Supplementary file 1 [file foods-13-02831-s001.zip › foods-3117175-supplementary.pdf]

Supplemental Table S1a: Analysis of sleepiness total by demographics

```
Call:
lm(formula = as.formula(paste(dep_var, formula_, sep = "~")),
    data = use)

Residuals:
    Min      1Q  Median      3Q     Max
-7.2118 -2.4936 -0.3177  1.8833 13.5640

Coefficients:
                                                   Estimate Std. Error t value Pr(>|t|)
(Intercept)                                          4.4389     0.8334   5.326 1.89e-07 ***
age266-75                                            0.1962     0.4251   0.462  0.64471
age2> 75                                            -0.4883     0.6767  -0.722  0.47111
genderMale                                           0.6542     0.3938   1.661  0.09764 .
race2Hispanic                                        0.1643     0.5910   0.278  0.78113
race2White                                          -0.6319     0.5717  -1.105  0.26989
race2other                                           0.2242     0.8176   0.274  0.78413
HH_income215,000 - 34,999                           -0.3951     0.6763  -0.584  0.55947
HH_income235,000 - 74,999                           -0.2509     0.6479  -0.387  0.69885
HH_income2>= 75,000                                  0.3831     0.6904   0.555  0.57938
highest_degree2some college/2 year or assoc degree  -0.3354     0.5410  -0.620  0.53568
highest_degree24 year or Bachelors degree           -0.8558     0.5883  -1.455  0.14673
highest_degree2Graduate degree                      -0.4266     0.6507  -0.656  0.51257
BMI_category2overweight (25-29.9)                    0.7078     0.4689   1.509  0.13215
BMI_category2obese (30+)                             1.1193     0.5092   2.198  0.02867 *
number_health_conditions_category2-3                 1.2302     0.4537   2.711  0.00706 **
number_health_conditions_category4+                  1.3245     0.5295   2.501  0.01287 *
---
Signif. codes:  0 ‘***’ 0.001 ‘**’ 0.01 ‘*’ 0.05 ‘.’ 0.1 ‘ ’ 1

Residual standard error: 3.464 on 321 degrees of freedom
Multiple R-squared:  0.09133,   Adjusted R-squared:  0.04604
```

F-statistic: 2.016 on 16 and 321 DF, p-value: 0.01194

Supplemental Table S1b: Analysis of Variance Table* for 1-way ANCOVA Analysis of Sleepiness Total

Model Term	Df	Sum Sq	Mean Sq	F-value	P-value
age2	2	13.210	6.605	0.551	0.577
gender	1	33.109	33.109	2.760	0.098
race2	3	42.152	14.051	1.171	0.321
HH_income2	3	27.782	9.261	0.772	0.510
highest_degree2	3	26.813	8.938	0.745	0.526
BMI_category2	2	60.580	30.290	2.525	0.082
number_health_conditions_category	2	108.782	54.391	4.534	0.011

*the F-tests assess the effect of the demographic variable after adjusting for the other demographics.

Supplemental Table S2a: Logistic Regression Analysis of Food Security categories by demographics

Note: food security status coded as 0=high or marginal, 1=low or very low.

```
Call:
glm(formula = as.formula(paste(dep_var, formula_, sep = "~")),
    family = "binomial", data = use)

Coefficients:
                                                   Estimate Std. Error z value Pr(>|z|)  
(Intercept)                                        -1.30799    0.64324  -2.033   0.0420 *
age266-75                                          -0.47591    0.32619  -1.459   0.1446  
age2> 75                                           -1.59169    0.79182  -2.010   0.0444 *
genderMale                                          0.40221    0.31012   1.297   0.1947  
race2Hispanic                                       0.38895    0.43475   0.895   0.3710  
race2White                                         -0.15856    0.44831  -0.354   0.7236  
race2other                                          0.49786    0.58065   0.857   0.3912  
HH_income215,000 - 34,999                          -0.10414    0.47965  -0.217   0.8281  
HH_income235,000 - 74,999                          -0.38832    0.47067  -0.825   0.4093  
HH_income2>= 75,000                                -0.87225    0.53997  -1.615   0.1062  
highest_degree2some college/2 year or assoc degree  0.89008    0.42737   2.083   0.0373 *
highest_degree24 year or Bachelors degree          -0.19650    0.50749  -0.387   0.6986  
highest_degree2Graduate degree                      0.54303    0.53574   1.014   0.3108  
BMI_category2overweight (25-29.9)                  -0.05017    0.36805  -0.136   0.8916  
BMI_category2obese (30+)                           -0.37552    0.41056  -0.915   0.3604  
number_health_conditions_category2-3               -0.31949    0.37300  -0.857   0.3917  
number_health_conditions_category4+                 0.55144    0.39912   1.382   0.1671  
---
Signif. codes:  0 ‘***’ 0.001 ‘**’ 0.01 ‘*’ 0.05 ‘.’ 0.1 ‘ ’ 1

(Dispersion parameter for binomial family taken to be 1)

    Null deviance: 322.10  on 317  degrees of freedom
```

Residual deviance: 287.14 on 301 degrees of freedom
AIC: 321.14

Number of Fisher Scoring iterations: 5

Supplemental Table S2b: Analysis of Deviance Table* for Analysis of Food Security Status*

Model Term	Df	Chi-Squared Statistic	P-value
age2	2	6.206	0.045
gender	1	1.687	0.194
race2	3	2.919	0.404
HH_income2	3	3.476	0.324
highest_degree2	3	9.640	0.022
BMI_category2	2	1.002	0.606
number_health_conditions_category	2	5.089	0.079

*the chi-squared statistics assess the effect of the demographic variable after adjusting for the other demographics.

Supplemental Table S3a: Linear Regression Analysis of Perceived Stress Total by demographics

```
Call:
lm(formula = as.formula(paste(dep_var, formula_, sep = "~")),
    data = use)

Residuals:
    Min      1Q  Median      3Q     Max 
-7.2118 -2.4936 -0.3177  1.8833 13.5640 

Coefficients:
                                                   Estimate Std. Error t value Pr(>|t|)    
(Intercept)                                          4.4389     0.8334   5.326 1.89e-07 ***
age266-75                                            0.1962     0.4251   0.462  0.64471    
age2> 75                                            -0.4883     0.6767  -0.722  0.47111    
genderMale                                           0.6542     0.3938   1.661  0.09764 .  
race2Hispanic                                        0.1643     0.5910   0.278  0.78113    
race2White                                          -0.6319     0.5717  -1.105  0.26989    
race2other                                           0.2242     0.8176   0.274  0.78413    
HH_income215,000 - 34,999                           -0.3951     0.6763  -0.584  0.55947    
HH_income235,000 - 74,999                           -0.2509     0.6479  -0.387  0.69885    
HH_income2>= 75,000                                  0.3831     0.6904   0.555  0.57938    
highest_degree2some college/2 year or assoc degree  -0.3354     0.5410  -0.620  0.53568    
highest_degree24 year or Bachelors degree           -0.8558     0.5883  -1.455  0.14673    
highest_degree2Graduate degree                      -0.4266     0.6507  -0.656  0.51257    
BMI_category2overweight (25-29.9)                    0.7078     0.4689   1.509  0.13215    
BMI_category2obese (30+)                             1.1193     0.5092   2.198  0.02867 *  
number_health_conditions_category2-3                 1.2302     0.4537   2.711  0.00706 ** 
number_health_conditions_category4+                  1.3245     0.5295   2.501  0.01287 *  
---
Signif. codes:  0 ‘***’ 0.001 ‘**’ 0.01 ‘*’ 0.05 ‘.’ 0.1 ‘ ’ 1

Residual standard error: 3.464 on 321 degrees of freedom
```

Multiple R-squared: 0.09133,   Adjusted R-squared: 0.04604
F-statistic: 2.016 on 16 and 321 DF, p-value: 0.01194

Supplemental Table S3b: Analysis of Variance Table* for 1-way ANCOVA Analysis of Perceived Stress Total

Model Term	Df	Sum Sq	Mean Sq	F-value	P-value
age2	2	13.210	6.605	0.551	0.577
gender	1	33.109	33.109	2.760	0.098
race2	3	42.152	14.051	1.171	0.321
HH_income2	3	27.782	9.261	0.772	0.510
highest_degree2	3	26.813	8.938	0.745	0.526
BMI_category2	2	60.580	30.290	2.525	0.082
number_health_conditions_category	2	108.782	54.391	4.534	0.011

*the F-tests assess the effect of the demographic variable after adjusting for the other demographics.

Supplemental Table S4a: 2-Way Factorial Food Security Status x Perceived Stress Category Analysis of Sleepiness Total, adjusting for demographic variables and Food Security Status

Call:
lm(formula = as.formula(formula_), data = use)

Residuals:

Min	1Q	Median	3Q	Max
-7.3308	-2.0469	-0.3568	1.5426	11.3815

Coefficients:

	Estimate	Std. Error	t value	Pr(>|t|)	
(Intercept)	2.98815	0.80445	3.715	0.000243	***
age266-75	0.22656	0.39978	0.567	0.571346	
age2> 75	-0.20875	0.64066	-0.326	0.744775	
genderMale	0.55864	0.37067	1.507	0.132843	
race2Hispanic	-0.21747	0.55228	-0.394	0.694034	
race2White	-0.86814	0.53042	-1.637	0.102757	
race2other	0.05863	0.75664	0.077	0.938291	
HH_income215,000 - 34,999	-0.02487	0.64348	-0.039	0.969198	
HH_income235,000 - 74,999	0.35805	0.62136	0.576	0.564891	
HH_income2>= 75,000	0.86077	0.66770	1.289	0.198352	
highest_degree2some college/2 year or assoc degree	-0.20698	0.50681	-0.408	0.683281	
highest_degree24 year or Bachelors degree	-0.42898	0.54642	-0.785	0.433032	
highest_degree2Graduate degree	-0.31080	0.60771	-0.511	0.609433	
BMI_category2overweight (25-29.9)	0.81351	0.43176	1.884	0.060522	.
BMI_category2obese (30+)	1.30165	0.48417	2.688	0.007587	**
number_health_conditions_category2-3	1.02911	0.42322	2.432	0.015626	*
number_health_conditions_category4+	1.09188	0.49004	2.228	0.026622	*
food_security_status2Very low or low food security	-0.95499	0.99457	-0.960	0.337736	
PSS_categorymoderate stress	1.35184	0.42219	3.202	0.001514	**
PSS_categoryhigh stress	3.40913	1.08606	3.139	0.001866	**
food_security_status2Very low or low food security:PSS_categorymoderate stress	2.61413	1.14527	2.283	0.023166	*

```
food_security_status2Very low or low food security:PSS_categoryhigh stress      1.99529    1.93008   1.034 0.302079
---
Signif. codes:  0 ‘***’ 0.001 ‘**’ 0.01 ‘*’ 0.05 ‘.’ 0.1 ‘ ’ 1

Residual standard error: 3.098 on 296 degrees of freedom
Multiple R-squared:  0.228,     Adjusted R-squared:  0.1732
F-statistic: 4.162 on 21 and 296 DF,  p-value: 1.196e-08
```

Supplemental Table S4b: Analysis of Variance Table* for Factorial Analysis of Sleepiness Total

Model Term	Df	Sum Sq	Mean Sq	F-value	P-value
PSS_category	2	283.547	141.773	14.767	0.000
food_security_status2	1	39.475	39.475	4.112	0.043
PSS_category:food_security_status2	2	50.024	25.012	2.605	0.076

*the F-tests for the 'main effects' of the factors (first 2 rows above) assess the effect of the factor after adjusting for the other factor and the demographic variables. The F-test in the last row assesses the interaction of the factors.

Supplemental Table S5: Linear regression model for Sleepiness Total as a function of Perceived Stress Total

```
Call:
lm(formula = doze_total ~ PSS_total, data = bh)

Residuals:
    Min      1Q  Median      3Q     Max 
-6.6839 -2.4543 -0.3177  1.7921 14.5138 

Coefficients:
            Estimate Std. Error t value Pr(>|t|)    
(Intercept)  2.92228    0.35978   8.122 6.63e-15 ***
PSS_total    0.18314    0.02253   8.129 6.32e-15 ***
---
Signif. codes:  0 ‘***’ 0.001 ‘**’ 0.01 ‘*’ 0.05 ‘.’ 0.1 ‘ ’ 1

Residual standard error: 3.34 on 376 degrees of freedom
Multiple R-squared:  0.1495,    Adjusted R-squared:  0.1472 
F-statistic: 66.09 on 1 and 376 DF,  p-value: 6.316e-15
```
